# Data on individual PCR efficiency values as quality control for circulating miRNAs

**DOI:** 10.1016/j.dib.2015.09.011

**Published:** 2015-09-26

**Authors:** Anna Brunet-Vega, Carles Pericay, María Elisa Quílez, María José Ramírez-Lázaro, Xavier Calvet, Sergio Lario

**Affiliations:** a Oncology Service, Hospital de Sabadell, Corporació Sanitària Parc Taulí, Institut Universitari Parc Taulí-UAB, Sabadell, Spain; b Digestive Diseases Service, Hospital de Sabadell, Corporació Sanitària Parc Taulí, Institut Universitari ParcTaulí-UAB, Sabadell, Spain; c Fundació Parc Taulí, Corporació Sanitària Parc Taulí, Institut Universitari ParcTaulí-UAB, Sabadell, Spain; d Centro de Investigación Biomédica en Red de Enfermedades Hepáticas y Digestivas (CIBERehd), Instituto de Salud Carlos III, Madrid, Spain

**Keywords:** Circulating microRNA, Quality control, Real time PCR, PCR efficiency

## Abstract

This data article contains data related to the research article entitled “Variability in microRNA recovery from plasma: Comparison of five commercial kits, doi:10.1016/j.ab.2015.07.018” Brunet-Vega (2015) [1]. PCR efficiency, along with RNA and cDNA quality, are the most important factors affecting the quality of qPCR results. Constant amplification efficiency in all compared samples is indispensable when relative quantification is used to measure changes in gene expression. An easy way to measure PCR efficiency, without the need of a standard curve, is LinRegPCR software. Individual PCR efficiency can be determined as a part of qPCR quality control. This is especially important when the initial RNA quantity is so low that cannot be accurately quantified, such as in circulating RNA extractions. This data article reports the Cqs and PCR efficiencies of 5 miRNAs quantified in RNA isolated from 4 patients with colorectal cancer (CRC) and 4 healthy donors using five commercially available kits.

**Specifications table**

**Table:** 

Subject area	*Biomedicine*
More specific subject area	*Circulating microRNA analysis*
Type of data	*Figures*
How data was acquired	*Data are Cq real-time PCR values and PCR efficiencies calculated with LinRegPCR software.*
Data format	*Analyzed data.*
Experimental factors	*CRC patients vs. healthy subjects.*
Experimental features	*miRNAs were extracted from plasma using five commercially available kits.*
Data source location	*Sabadell, Barcelona, Spain.*
Data accessibility	*Data found in this article*

**Value of the data**

• Data presented here shows that determining PCR efficiency for every miRNA amplicon in a small pilot study is useful to improve the design and interpretation of subsequent larger experiments.
• We show the use of LinRegPCR for the detection of amplicons with suboptimal PCR efficiency. Amplicons with poor performance may need optimization or new primer design.
• We show that individual samples behaving differently can be detected.
• We show the effect of assay design on PCR efficiency, Cqs and variability. Increasing PCR efficiency leads to a reduction in Cqs and variability.
• We show that some miRNA assays have low PCR efficiency and therefore qPCR results have to be interpreted with caution.

## 1 Data

**Fig. 1:**
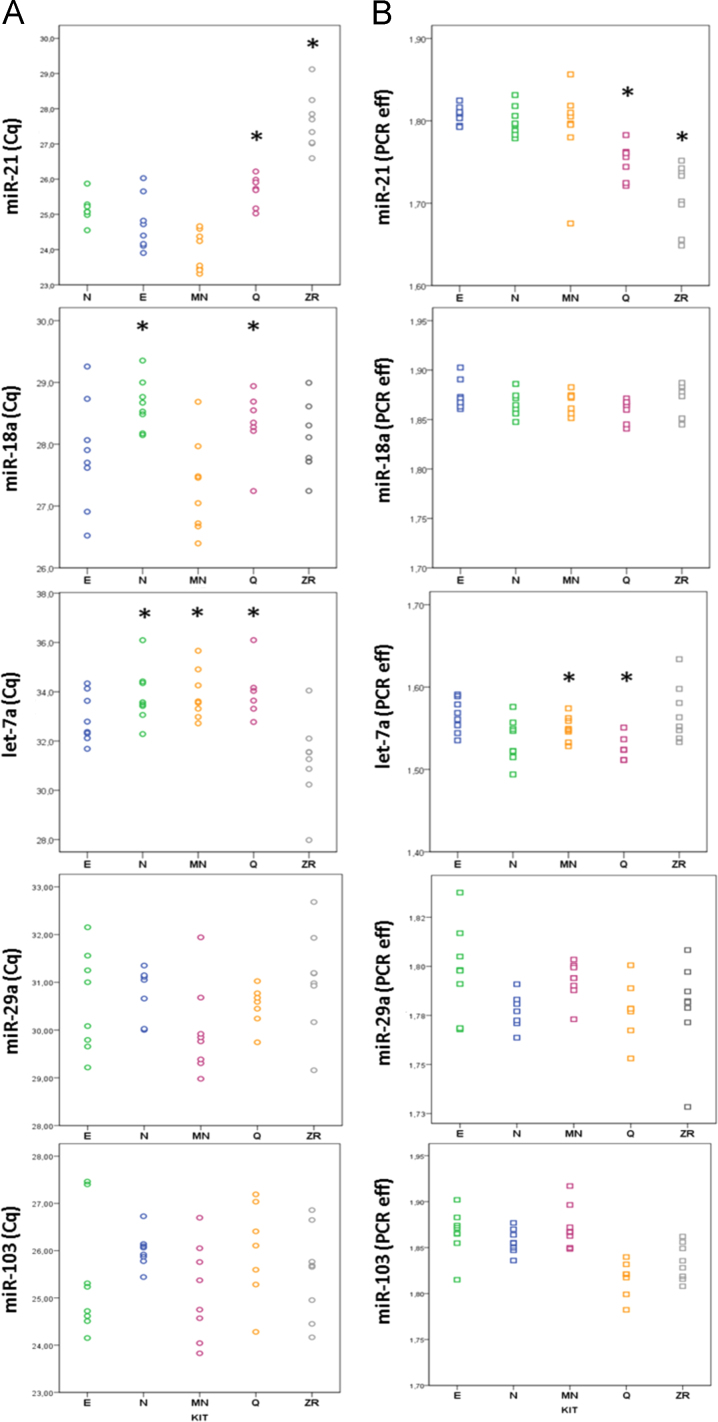
Cq (A) and PCR efficiency (B) for miRNA-21, miRNA-18a, let-7a, miR-29a and miR-103 in the eight samples analyzed. The Cqs and PCR efficiencies of miR-103 and miR-29a did not differ between the 5 extraction methods (*p*=ns, one-way ANOVA). However, the relative expression levels for miR-21, miR-18a, and let-7a kits differed depending on the kit used. For miR-21, the mean Cq for the MN kit was 23.9±0.20, which was 1.7 to 3.6 higher than the mean Cq for kits Q and ZR (*p*<0.05, one-way ANOVA). The lower Cq values for kits Q and ZR can be attributed to their lower PCR amplification efficiency for miR-21 (*p*<0.05, one-way ANOVA). For miR-18a, Cq values were slightly lower for kits N and Q (*p*<0.05, one-way ANOVA). For let-7a, PCR efficiency was suboptimal (1.54±0.008); Cq values for let-7a were lower (>2Cqs) for kit ZR (*p*<0.05, one-way ANOVA).

**Fig. 2:**
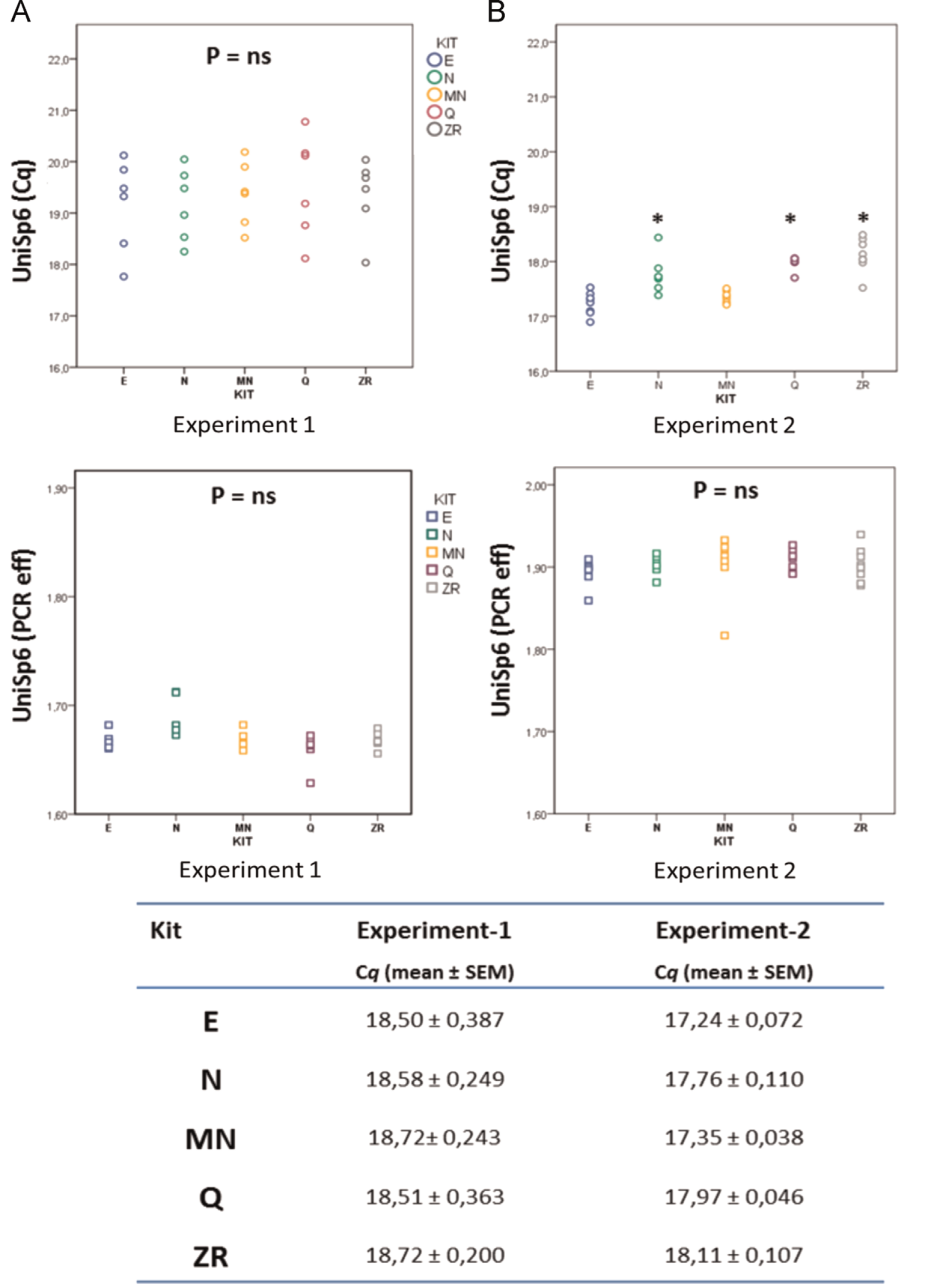
The impact of PCR efficiency on Cqs. Exiqon redesigned the UniSp6 spike-in [2]. The graph shows the Cq (A) and PCR efficiency (B) of the original design (*experiment 1*) and the new design (*experiment 2*). The improvements resulted in a higher PCR efficiency (1.67±0.015 vs 1.90±0.02) that resulted in lower Cq values and variability (C). * *p*< 0.05, respect to E and MN.

**Fig. 3:**
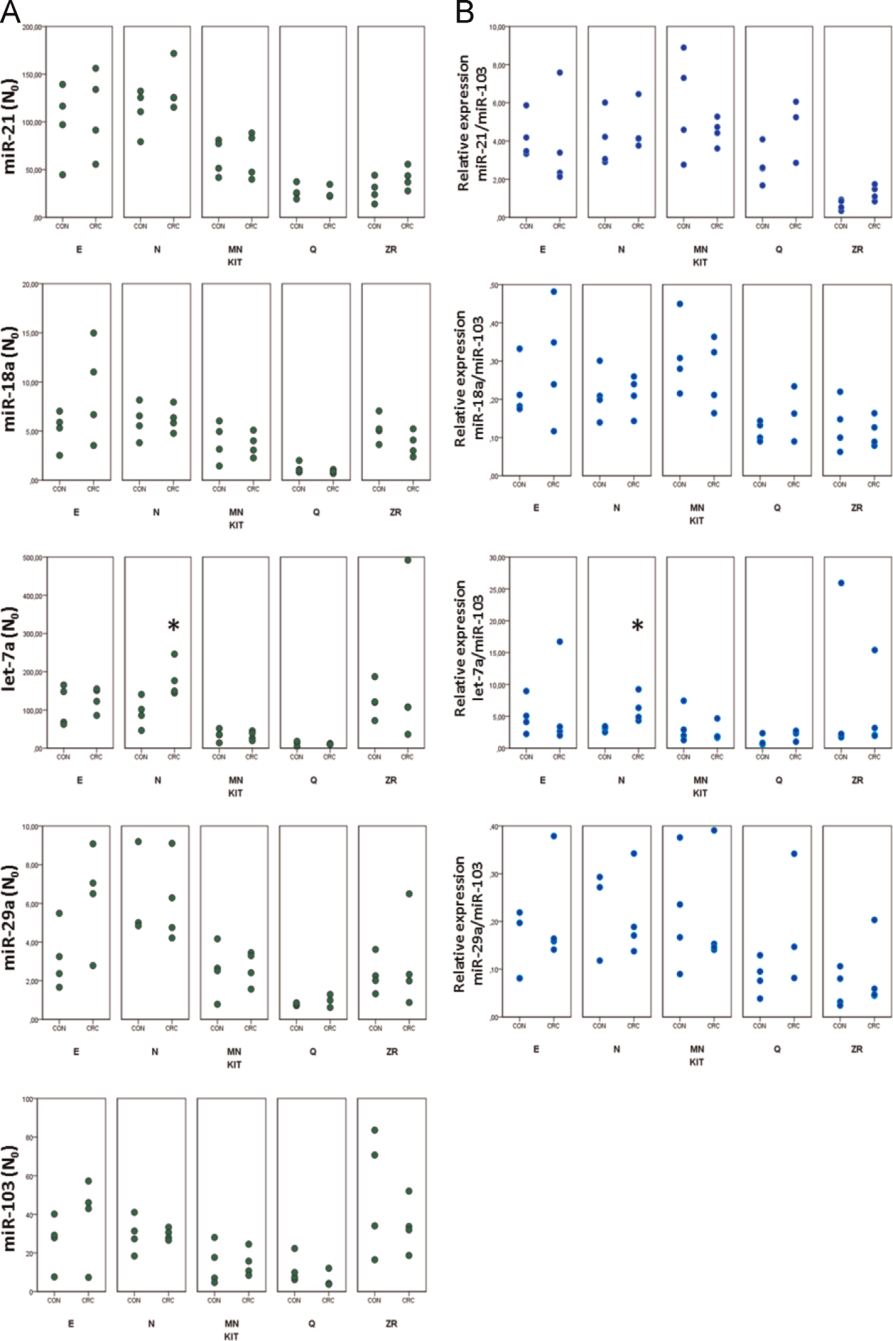
Comparison of miRNA expression in CRC patients and healthy controls. (A) shows the corrected *N*_0_ value and (B) shows the relative expression of candidate miRNAs corrected by the endogenous miRNA miR-103. Significant differences in let-7a expression were found between controls and CRC patients for kit N. Caution must be taken to interpret this result because let-7a displayed low PCR efficiency (1.53±0.009) together with high Cqs (33.8±0.40). We found no significant differences between controls and patients for the other miRNAs analyzed. * *p*< 0.05, two-tailed *t*-test. The *N*_0_ values were corrected for the initial plasma volume used for the RNA extraction and for the elution volume according to the kit׳s instructions. Abbreviations are: CRC, colorectal cancer patients; CON, healthy controls.

The data presented in this article show the plots of the PCR efficiency and Cqs of different miRNA assays. Data was obtained by extracting plasma RNA from 8 patients (4 cancer and 4 healthy) using five commercially available kits (Figs. 1–3).

**Table:** 

miRNA-21,	*p*<0.05 respect to N, E, and MN kits.
miRNA-18a,	*p*<0.05 respect to E, MN, and ZR kits.
Let-7a,	*p*<0.05 respect to E and ZR kits.

## 2 Experimental design, materials and methods

### 2.1 Material and methods

We isolated miRNAs in plasma from colorectal cancer patients (stage IV) and healthy donors with five commercially available kits (Exiqon, Norgen, Macherey-Nagel, Qiagen, and Zymo Research). After isolating RNA with the five kits, we measured the abundance of four candidate miRNA biomarkers for colorectal cancer (miR-21, miR-18a, let-7a, and miR-29a) and miR-103 as endogenous control. Details about RNA extraction, cDNA synthesis and qPCR can be found in [2]. Raw data were obtained from QuantStudio 6 and 7 Flex Real-Time PCR System Software (Applied Biosystems, Foster City, CA, USA), exported in rdml format [3], and imported to LinRegPCR (Heart Failure Research Center, Amsterdam, the Netherlands) [4,5]. Using an iterative algorithm, LinRegPCR determines baseline fluorescence, sets a window of linearity (W-o-L) for each amplicon, and calculates the PCR efficiency (E) per sample and amplicon. The algorithm also calculates the Cq value and the starting concentration per sample (*N*_0_) using the formula *N*_0_=*N*_q_/*E*^Cq^, where *N*_q_ is the fluorescence threshold set to determine Cq. Individual PCR efficiencies and Cq were analyzed using SPSS v21 (IBM Corporation, Armonk, NY, USA). To determine whether circulating miRNAs were normally distributed, we used Q-Q normal plots. We used Levene׳s test for the homogeneity of variances. We used one-way analysis of variance (ANOVA) with Tukey post-test or two-tailed *t*-test, as appropriate. Significance was set at *p*<0.05.

## Competing interests

None.
